# Food Insecurity and Dietary Intake among Rural Indian Women: An Exploratory Study

**DOI:** 10.3390/ijerph18094851

**Published:** 2021-05-01

**Authors:** Alice Sims, Paige van der Pligt, Preethi John, Jyotsna Kaushal, Gaganjot Kaur, Fiona H McKay

**Affiliations:** 1 The School of Health and Social Development, Deakin University, Geelong, VIC 3220, Australia; aasims@deakin.edu.au; 2 Institute for Physical Activity and Nutrition (IPAN), School of Exercise and Nutrition Sciences, Deakin University, Geelong, VIC 3220, Australia; p.vanderpligt@deakin.edu.au; 3 Chitkara School of Health Sciences, Chitkara University, Punjab 144417, India; preethi@chitkara.edu.in; 4 Centre of Water Sciences, Chitkara University Institute of Engineering and Technology, Chitkara University, Punjab 144417, India; jyotsna.kaushal@chitkara.edu.in; 5 Chitkara Business School, Chitkara University, Punjab 144417, India; gaganjot.kaur@chitkara.edu.in

**Keywords:** food security, India, women, rural

## Abstract

Food insecurity is an important contributor to health and a factor in both underweight and malnutrition, and overweight and obesity. Countries where both undernutrition and overweight and obesity coexist are said to be experiencing a double burden of malnutrition. India is one example of a country experiencing this double burden. Women have been found to experience the negative impacts of food insecurity and obesity, however, the reasons that women experience the impact of malnutrition more so than men are complex and are under-researched. This current research employed a mixed methods approach to begin to fill this gap by exploring the dietary intake, anthropometric characteristics, and food security status of rural Indian women. In total, 78 household were surveyed. The average waist measurement, waist to hip ratio, and BMI were all above WHO recommendations, with two thirds of participants categorized as obese. Contributing to these findings was a very limited diet, high in energy, and low in protein and iron. The findings of this research suggest that the rural Indian women in this study have a lack of diet diversity and may be at risk of a range of non-communicable diseases.

## 1. Introduction

Increased global wealth and access to healthcare means that over the last century, many people are living healthier lives for longer [1,2,3]. These gains, however are not universal, with many low- and middle-income countries (LMIC) seeing increased prevalence of obesity and nutrition-related noncommunicable disease (NCD) [2]. NCDs, including cancers, cardiovascular diseases, chronic respiratory diseases, and diabetes are the leading cause of ill health worldwide and result in almost three quarters of global deaths each year [2].

Poor nutrition is a significant contributor to poor health; it can increase the risk of NCDs, obesity and obesity related morbidity, and can markedly reduce life expectancy [1]. Food security is defined a state that exists when ‘all people, at all times, have physical and economic access to sufficient, safe, and nutritious food that meets their dietary needs and food preferences for an active and healthy life’ [4], the absence of food security, referred to as food insecurity, can increase the risk of malnutrition and a range of poor health outcomes [5,6]. Food insecurity is increasingly recognized as an important contributor to health and is a significant component in the development and progression of NCDs. While malnutrition is generally associated with undernutrition (wasting, stunting, underweight), it is increasingly recognized as a consequence of overnutrition, playing a role in both the development and progression of diet-related NCDs [7].

Settings where undernutrition and overweight and obesity coexist are said to be experiencing a double burden of malnutrition. India is one example of a country experiencing this double burden [8]. Like many LMICs, India is experiencing increasing rates of NCDs, accounting for over half of annual deaths [9]. Women are more likely to be impacted by this double burden and are more likely to have an NCD and experience overweight or obesity [10,11]. While once known for high rates of stunting and wasting, global trends predict that by 2040 the overall prevalence of overweight in India will be 27.8% and that 5% of the population will be obese [12,13]. The reasons that women experience the impact of malnutrition more so than men are complex. Some research suggests that it may be related to inequalities in education [14], employment [15], or entitlement to land [16], however, little research has explored the experiences of food security and diet among women in India to understand the lived experience of this double burden. This research seeks to fill this gap by employing a mixed methods approach to explore the dietary intake, anthropometric characteristics, and food security status of rural Indian women.

## 2. Method

This research employed a mixed method approach to explore obesity, dietary intake, and food security status among women in India. The comprehensive study protocol for this study can be found elsewhere [17]. The main aims of this research are to determine the anthropomorphic status; and investigate the dietary patterns, food habits, and food security status of women. Ethics approval was granted by the Deakin University Research Ethics Committee (2020-007) and the Institutional Ethics Committee of Chitkara University (IHEC/DHR/CU/PB/20/14).

### 2.1. Sample and Recruitment

Participants were recruited from a village consisting of 500 dwellings, in the Punjab region of north India (see Supplementary Materials for location). Before recruitment began, the village Sarpanch, the official elected head of the village, was contacted to discuss the research and recruitment strategy. The research team consisted of researchers local to the area and researchers from Australia. The region is largely agricultural and, as a result, some village members were away at the time of data collection, as such it was determined that a convenience sample was most appropriate given the exploratory nature of the research and the uncertainty of dwelling occupation. At the beginning of data collection, the village was divided into two halves and two research teams commenced door knocking households. Local trained researchers who were fluent in Punjabi, Hindi, and English worked as translators for the period of data collection. Informed consent was obtained from all participants to prior to data collection.

### 2.2. Data Collection

Face-to-face structured interviews were conducted at a place of the participants preference, typically in a private home. Households of any size and type were included. Interviews included four components.

1. Demographic questions including age of participant, the age and sex of the head of household, the religion of the household, household structure, and approximate monthly income of the household;
2. Anthropomorphic measurements including height, weight, and body measurements of the main women of the house, typically the wife of the head of the household. All participants were weighed without shoes to the nearest 0.1 kg using a portable, calibrated Tanita digital scale. Height measurements were taken with a Seca 213 portable stadiometer to the nearest 0.5 cm. Waist circumference was taken with a Seca tape measure in centimeters at the midway point between costal margin and iliac crest in the mid-axillary line, with participant standing neutrally and breathing normally. Hip circumference was measured at the widest point of the greater trochanter in centimeters. All measurements were taken twice, and the average is used;
3. Additionally, 24-h dietary recalls were used to collect data on food and beverage consumption over the previous 24-h period. Portion sizes were explained in spoon- or “hand-full” or by showing the researcher the dishes they would eat from. Recipes of frequently consumed meal types were provided during the interviews;
4. Food security status was collected via the 4-item version United States Department of Agriculture, Household Food Security Survey Module, developed by Blumberg and Bialostosky [18], along with questions relating household sources food, how often they eat, if they consider their eating patterns to be different now than they were in the past, and who makes household decisions.

All households were visited once; where timing permitted, a selection of households was visited to complete a second 24-h diet recall. Interviews lasted between 30–60 min, were audio recorded and supplemented with detailed written notes. Interviews were conducted in Punjabi with one or two translators present to provide simultaneous translation into English, to ask further questions or for additional probing.

Due to the COVID-19 pandemic, data collection was suspended two weeks earlier than planned. The original aim was to collect data from 100 households, however, the global pandemic and the requirement for the Australian researchers to leave India on 18 March 2020 and widespread lockdowns in India, meant fewer than planned interviews and duplications of the 24-h dietary recall could be collected.

### 2.3. Data Analysis

Basic descriptive statistics were computed for demographic data, including means and standard deviations. Data from 24-dietary recall data were analyzed using Indian food composition tables compiled by the National Institute of Nutrition [19]. These tables comprise lists of nutritional values specifically for Indian grown produce and were used to establish accuracy in the nutrient content of foods. For comparison, secondary analysis was performed using established nutrition software FoodWorks version 10 (Xyris 2019). FoodWorks provides largely ‘Western’ diet composition data (Xyris Software\FoodWorks; Xyris Software, Highgate Hill, Australia). A number of pre-existing and suitable recipes were available in the database, while others needed to be created using the recipes provided by participants. Both sets of analysis provided totals for energy in kilojoules (KJ), protein, fat, saturated fat, carbohydrate, fiber in grams (g), and sodium and iron in milligrams (mg). These totals were then analyzed to calculate the mean and standard deviation; comparisons were made with Indian recommended daily allowances (RDA).

Body mass index (BMI) was calculated as weight (kg) divided by height (m) squared (kg/m^2^). Criteria adopted by Indian standards were used to define obesity as BMI ≥ 25 kg/m^2^ [20]. Waist to hip ration was calculated as the measurement of waist divided by hip as recommended by WHO [21]. The lower recommended waist circumference, that is a cut-off point of 0.80 was used as per the guidelines for Asian populations who are at greater health risk at lower measurement than Europeans [21].

In accordance with the method proposed by Coleman-Jensen, Gregory, and Singh [22], the food security scores were combined to create one measure for level of food security for a household. The food security status of each interviewed household is determined by the number of food insecure conditions and behaviors that the household reports. Households with zero or one affirmative response are classified as food secure, those with two or more affirmative responses are classified as food insecure and those with three or more affirmative responses are classified as food insecure with hunger. Data were analyzed using SPSS, version 25.

## 3. Results

### 3.1. General Characteristics

In total, 78 household surveys were completed. The demographic characteristics are presented in Table 1. Almost all households were Sikh (*n* = 76, 97.5%), most of the participants were female (*n* = 73, 93.5%), while most of the heads of household were male (*n* = 53, 68%). The average age of participants was 45 years (SD = 15.6, range 18 to 94), younger than the average age of the heads of the household which was 58 years (SD = 13.1, range 30 to 95). Most households were joint (*n* = 53, 68%), and over half of the households interviewed had six or more residents (*n* = 40, 51.3%), around three quarters of households had at least one child under the age of 18 (*n* = 57, 79%). Over two thirds (*n* = 53, 68%) of households had some land that they are able to farm, with most respondents indicating that their main crop was wheat, followed by potatoes and other vegetables. Almost one half of households had at least one member who consumed any alcohol (*n* = 37, 47.4%), and only 3 households had a household member who consumed tobacco. Households reported an average monthly income of Rs. 20,191(1 USD = 73 Rs at 10 March 2021) (SD 22,123; range 5000 to 150,000), and almost half (*n* = 34, 43.5%) made decisions about household spending as a family.

When asked how the food they consume today compared to that of previous generations, most participants (*n* = 60, 77%) reported that the food of today is less healthy. This was largely related to the presence of pesticides used in farming, although some participants were concerned about the amount of fast food consumed by younger generations. Many of these participants said that previous generations were more likely to prepare their food at home, contributing to the healthiness of the food.

Of the 53 households who responded to the food security questions, only 5 (9.4%) were identified as food insecure. However, diet diversity (see below) suggests that while households are technically food secure according to the measure employed by this research, they have limited variety in their diet and may be vulnerable to shocks.

### 3.2. Andromorphic Data

Weight, height, and waist and hip circumference measurements were collected from 42 female participants. The average waist measurement was 88.4 cm (SD 12.3), higher than the WHO recommended healthy waist measurement of 80 cm or less. The average waist to hip ratio was 0.90 (SD 0.11), above the WHO recommendation of 0.80 or less. The average BMI was 27 kg/m^2^, above the WHO recommendation of a health BMI (22.9 kg/m² or less). Over one third of all participants (*n* = 16, 38.1%) were in the obese category I as classified by the WHO, while a third were categorized as obese category II (*n* = 11, 26.1%). The remainder were divided between pre-obese (*n* = 6, 14.4%) and healthy (*n* = 9, 21.4%). Table 2 shows the anthropomorphic characteristics of the sample.

### 3.3. Nutrition Data

Table 3 and Table 4 below present the findings from the nutritional analysis. Data for this study were analyzed in two ways, allowing for a comparison of results between data analyzed according to the Indian food composition tables (IFCT) with the data analyzed according to the nutritional software FoodWorks v10.0, more commonly used in high income settings. The IFCT consist of a list of nutritional values specific to Indian grown produce. Use of these two data sources for composition analysis is useful here as there are times when the IFCT are incomplete, using the FoodWorks data provides a point of comparison with a thorough ingredient list. Two days of 24-h dietary recall data were included for 28 participants. Results are presented as mean values over the two days.

Table 3 shows that, on average women met 72.6% of the recommended energy intake, consuming on average 5774.9 kj; below the recommendation of 7950 kj per day. On average, protein intake and iron intake were lower than recommendations while fat and sodium daily intakes were above recommendations. 

The 24-h diet recall data revealed that most women in the village consume a diet that consists of several food groups that repeat across meals. The most common food reported was chapati (homemade wheat bread) followed by potato curry, chai (tea) with milk and sugar, and a dairy drink of buttermilk. Other foods less frequently reported included rice, dahl and pulses, fried bread, such as paratha, curd, Indian sweets, and, very infrequently (only a few households), fruits such as grapes or oranges. Overall, the 24-h diet recall demonstrated a lack of diversity, with protein and other nutrient rich sources lacking. Foods such as pulses, green vegetables, fruits, seeds, and nuts were eaten very infrequently.

Table 4 presents the results of data analyzed using FoodWorks software. The purpose of this analysis was to gain an understanding of what the dietary patterns looked like using a ‘Western style’ food database. While this provided an interesting comparison, the results were less likely to be accurate as the ingredients are based on Australian foods. When comparing these results, it can be noted that according to FoodWorks, women were consuming more energy, more fat, more saturated fat, and more fiber, with lower estimates of carbohydrates and sodium. This is a reflection of both the incomplete nature of the Indian food composition tables and the inappropriateness of using a Western database like FoodWorks for an analysis of Indian food data. While the most accurate results likely fall between these values, without more specific analysis of the foods and composition patterns of Indian women, these values remain uncertain.

## 4. Discussion

This research sought to explore the dietary intake, anthropomorphic characteristics, and food security status of a sample of rural Indian women. This study found very high rates of overweight and obesity and a diet consisting of high amounts of nutrients, which have been shown to contribute to NCDs such as total fat and sodium well in excess of recommendations. Interconnected factors surrounding agricultural changes, the traditional and emerging roles of women in India, and the ongoing nutrition transition are key in understanding and contextualizing the results of this study.

### 4.1. Agriculture and Health in India

Changes to dietary patterns over the past five decades have been suggested as playing a role in emerging diet related diseases in India [23]. Responding to hunger and significant and widespread undernutrition, the Indian food system underwent major changes in the 1970s. These changes, now referred to as the Green Revolution, consisted of an overhaul of the agricultural system with the aims of increasing food production thanks to the adoption of modern technologies and methods. Through the introduction of high yield variety crops, the Green Revolution resulted in increased grain production, easing the burden of hunger for millions within a decade [23]. The epicenter of this development was the Punjab region, known as India’s bread belt, where high yield variety crops and high-input–high-output cropping techniques transformed agricultural output [16]. Wheat and rice were the main emphasis for production at this time, with more nutritious and low-glycemic index grains such as millet almost completely removed from production [23]. The intensity of the agriculture and largescale use of chemical fertilizer meant that over time, the soil nutrient content depleted faster than it could be replenish, also decreasing the nutritional content of the foods grown [23]. The ongoing impacts of this intensive agriculture were evident in the diets and lifestyles of participants of this study who were consuming mainly ground wheat in the form of chapati wheat bread, with few other nutritious grains or vegetables evident in their diet, playing a significant role in their ongoing health and wellbeing [24].

In addition to the limited dietary diversity resulting from current agricultural practices, farmers in the Punjab region rely on increasing amounts of fertilizer, new seeds, and pesticides to make up for soil depletion [25]. The increasing costs of these products and the need to constantly use them can be a significant financial burden for households, with many forced to seek unregulated loans to cover their costs [26]. Farming households, including those in the present study, can experience unexpected financial shock such as crop failure due to changing weather, leading to financial hardship and increase food insecurity [27]. Households in this study were often large, likely spending half, or more, of the monthly income on food, leaving inadequate funds for household expenses and financial security. While only five households in this study were identified as food insecure, the lack of dietary variety observed suggests that these households are vulnerable to financial shock and food insecurity. 

A lack of dietary diversity has been linked to obesity and subsequent poor health outcomes [1]. Many families included in this study grow wheat crops and some vegetables for both income and their own grain supply. However, while many reported growing vegetables, only small amounts of vegetables were consumed. A homegrown diverse vegetable supply relies on available farmland, labor, sufficient funds for fertilizers and seeds, seasonality, and favorable weather patterns.

### 4.2. The Changing and Evolving Role of Women in India

Rural villages, like the one in this study have seen recent increase in the outmigration of men seeking livelihood opportunities in cities. This is referred to as the feminization of agriculture, where women are forced to take farming roles traditionally the responsibility of men [28]. Women in these roles are unlikely to have ownership and control over land, a factor in the enhancement of livelihoods of rural communities [28]. The agricultural roles held by women are often poorly- or unremunerated and their contribution is often invisible and unofficial [29]. Women have long been discriminated in land ownership in the Punjab region where property and wealth are typically passed down the male lineal descendant [30]. These norms of land ownership are a tangible representation of the low social power and position held by many women in rural communities. Across India, only 13.5% of land titles are in the names of women and in the Punjab region, this figure is just 0.9% [28].

With increased agricultural tasks women could be more in control of food and farming resources, which could help to promote the wellbeing of the woman and her family which would make them less susceptible to developing NCDs or obesity [26]. However, the relationship between increased contribution to farming and women’s empowerment is not necessarily a positive one. For many women in rural villages, the addition of farm work decreases their available time and detracts from opportunity for personal growth and empowerment. In India, agriculture forms 54.6% of all labor and roughly one third of farm laborers are women [28]. As observed in this study, women are typically responsible for household duties, however, of the 42 households interviewed for this study only 15 (35.7%) reported having a female head of house. This is consistent with longstanding patriarchal systems in Indian society, where gender inequality and discrimination of women are still widespread, especially in rural areas [26]. Some research suggests that by paying women for their workforce contribution, the economic participation would increase India’s overall GDP by 20% [31]. If women’s existing agricultural participation gained formal status, then she may be empowered to take control over household decision-making, increasing household food security, resulting in better health for the woman and her family [26]. Evidence suggests that when mothers are able to control financial resources they choose to allocate more to nutrition, education, and wellbeing [32]. Educating women and girls about healthy nutrition has a ripple effect in the community and helps to prevent the spread of overweight, obesity, and NCDs [29].

A lack of autonomy was clear during the interviews with the women of this study. At times women were unable to answer basic questions about household income and expenditure. During these instances the woman deferred to her husband or son if they were present. These moments pass unnoticed in the interview and appear to be accepted cultural norms. This finding is consistent with literature that demonstrates that women are reliant on their marriage for financial security and the security of their children, they are bound to the patriarchal system in which they have been born [28]. Marriage is vital to a women’s survival in India where heterosexual partnerships are the norm [33]. All the women interviewed for this study were married and almost all had at least two children, some had three or four. The majority of the women reported that their own mothers were illiterate or educated to the fifth or eighth grade and most of the women had received less education than their husbands. Closing the gender gap has become a global priority and is highlighted in sustainable development goal number 5 which aims to give women equal rights to economic resources, ownership and control over land, inheritance, and financial services [34]. While most of the women had active roles in family agriculture, they reported their own occupation as *housewife*. To address the growing issue of obesity and NCDs, women’s socio-political power must be enhanced. Education, a removal of cultural expectations and equal opportunity to land ownership are vital [16].

### 4.3. Changing Food Habits and the Nutrition Transition

The epidemiological transition seen across in India is largely attributable to changes in long held dietary patterns, a change that has been said to be responsible for the increasing rates of overweight, obesity, and NCDs [35]. Through globalization, changes to agriculture and a shift to more ‘Western’ style foods, diets are rapidly changing, and the country is now in what is referred to as in a state of a nutrition transition [36]. Urgent action is needed to intercept this public health issue and its impact on rapidly increasing rates of NCDs across India. Between 1998 and 2015, overweight across India increased in women from 8.4% to 15.5% and obesity from 2.2% to 5.1% [13]. Today in the Punjab region, 29.9% of women and 22.2% of men are obese [35]. These rates are consistent with the results of this study where over a third of all participants (38.1%) were classified as obese category I according to the WHO, a further third were categorized as obese category II (26.1%), and the remainder were divided between pre-obese (14.4%) and healthy (21.4%).

A traditional diet, once diverse and filled with wholegrain cereals now includes more animal-based products, refined oils, saturated fats, and refined sugars (Aiyar et al. 2021). In addition to these dietary changes, lifestyles have also changed as many physical forms of labor have become mechanized, increasing sedentary hours [36]. Socio-economic developments and increased affluence also influence health outcomes and the rate of epidemiological transition. The wealthier states of Kerala, Tamil Nadu, and Punjab have some of the highest rates of NCDs [32]. With increased wealth comes the affordability of luxury foods, the employment of staff and often sedentary hours are increased [12].

Almost half of the Indian population continue to follow a traditional vegetarian diet [37]. A complete vegetarian diet should consist of a variety of nutrient rich pulses and wholegrains, dairy products, iron rich leafy greens, fruit, nuts and seeds in order to meet iron, zinc, and other nutrient requirements [33]. Women included in this study were mostly vegetarian, however intakes of protein and fiber were lacking in their diets, likely attributable to the high intake of chapati wheat bread and relatively low intake of wholegrains and pulses. Given these families produce their own wheat crops, it is not surprising that wheat consumption is high and that foods, such as fresh fruit and vegetables, which are more expensive, were consumed less. With almost half of Indians consuming a vegetarian diet, the epidemiological trend of obesity and NCDs might seem contradictory as a vegetarian diet is often promoted for its ability to protect from NCD risk factors such as obesity, high blood cholesterol, and increased blood sugar [37]. However, the traditionally high carbohydrate component of the Indian diet, as observed in this study, and the relative absence of nutrient dense vegetables should be considered as potential contributors to the high rates of obesity and a variety of NCDs [36]. During interviews, older participants reported enjoying a more traditional vegetarian diet and the younger generation preferred more commercial food products available in the neighboring villages. Sodium intake of the women in this study was almost double the recommendation and presents an increased risk of contributing to hypertension in the short and long term. The addition of salt to a nutrient poor meal will give it interest, however if women are not aware of the health risks associated with excess salt intake, salt may be used in excess during meal preparation. These traditional dietary practices highlight the challenges India faces in coming years with nutrition and the burden of NCDs as younger generations transition to a diet higher in salt and sugar and away from a traditional diet.

## 5. Conclusions

Overweight or obesity was common in our sample of rural women living in India which is concerning, and consistent with evidence from many other LMIC settings [8]. Diet diversity was lacking, especially in fruits and vegetables, and intakes of total fat and sodium were high which is also concerning, given the well-established association of unhealthy eating behaviors and onset of nutrition-related chronic disease. Prevalence of overweight and obesity among women alongside sub optimal dietary intakes present a challenge for women in India and the public health response more broadly. While small and not seeking to be a representative sample, this study has contributed important knowledge regarding NCD-related risk factors for women living in rural India and further work should focus on exploring potential drivers of overweight and obesity and unhealthy dietary intakes among such populations. A multitude of complex factors needs to be considered in successfully implementing targeted approaches to reducing rates of NCDs across India.

## Figures and Tables

**Table 1:** Demographic characteristics, *n* = 78.

	*n* (%)
**Sex of Participant**	
Female	73 (93.5)
**Age of Participant**	
20–39	29 (37.1)
40–59	32 (41.0)
Over 60	14 (17.9)
Missing	3 (3.8)
**Sex of Head of Household**	
Female	25 (32.0)
**Age of Head of Household**	
20–39	6 (20.5)
40–59	31 (39.7)
Over 60	41 (52.5)
**Religion**	
Sikh	76 (97.5)
Hindu	2 (2.5)
**Family Type**	
Joint	53 (68.0)
Nuclear	25 (32.0)
**Number of People in House**	
1–2	5 (6.4)
3–5	33 (42.3)
6+	40 (51.3)
**Number of Children under 18**	
0	21 (26.9)
1	15 (19.2)
2	21 (26.9)
3	8 (10.2)
4+	13 (16.7)

**Table 2:** Anthropomorphic characteristics (*n* = 42).

	*n* (%)
**BMI Category**	
Normal (18.5–22.9)	9 (21.4)
Pre-obese (23–24.9)	6 (14.4)
Obese I (25.0–29.9)	16 (38.1)
Obese II (30–34.9)	11 (26.1)
**Waist and Hip Measurements**	
Waist (cm)	88.42 (12.34)
Hip (cm)	98.01 (8.74)
WH Ratio	0.90 (0.11)
**WH Ratio Category**	
Low (<0.80)	7 (17)
Moderate (0.81–0.85)	13 (31)
High (>0.86)	22 (52)

**Table 3:** Mean daily intakes using Indian food composition tables and comparison with Indian Council of Medical Research recommendations * (*n* = 42).

Nutrient Intake per Day	Mean (SD)	Range	Recommended	% of Recommended Intake
Energy (kj)	5774.9 (1546.0)	4750.25–9242.4	7950 kj *	72.6
Protein (g)	49.8 (20.4)	39.85–72.6	55 g *	90.5
Fat (g)	35.4 (17.6)	21.0–75.0	20–25 g *	157.3
Saturated Fat (g)	3.4 (4.4)	0.1–16.5	<7 g	48.5
Carbohydrate (g)	267.9 (75.9)	210.9–442.7	N/A	–
Fibre total (g)	18.0 (6.4)	13.5–33.1	>25 g	72
Sodium (mg)	3605.1 (1180.4)	2711.4–5777.9	<2000 mg ^	180.2
Iron (mg)	13.9 (27.3)	6.0–16.2	21 mg *	66.1

* Longvah et al. (2017); ^ WHO (2012), N/A (not applicable).

**Table 4:** Mean daily intakes using FoodWorks (*n* = 42).

Nutrient Intake/Day	Mean (SD)	Range
Energy (kj)	6670.7 (1837.3)	5654–11,170
Protein (g)	49.4 (13.7)	41–80.1
Fat (g)	50.5 (16)	38.9–80.6
Saturated Fat (g)	16.2 (9.5)	9.3–64.6
Carbohydrate (g)	223.3 (67.9)	180.5–397.0
Fibre total (g)	24.6 (7.5)	20.3–45.4
Sodium (mg)	2125.6 (609)	1799.5–3607.2
Iron (mg)	11.6 (4.3)	8.6–22.1

## Data Availability

The data presented in this study are available on request from the corresponding author. The data are not publicly available due to ethical consideraions.

## Supplementary Materials

The following are available online at https://www.mdpi.com/article/10.3390/ijerph18094851/s1. Figure S1: study location.

## Abbreviations

BMI	Body Mass Index
FAO	Food and Agriculture Organization
WHO	World Health Organization
