# Supplementary figures and images for: Food Insecurity and Dietary Intake among Rural Indian Women: An Exploratory Study
Source: Int J Environ Res Public Health. 2021 May 1;18(9):4851. doi: 10.3390/ijerph18094851 (PMC8124183; doi:10.3390/ijerph18094851)

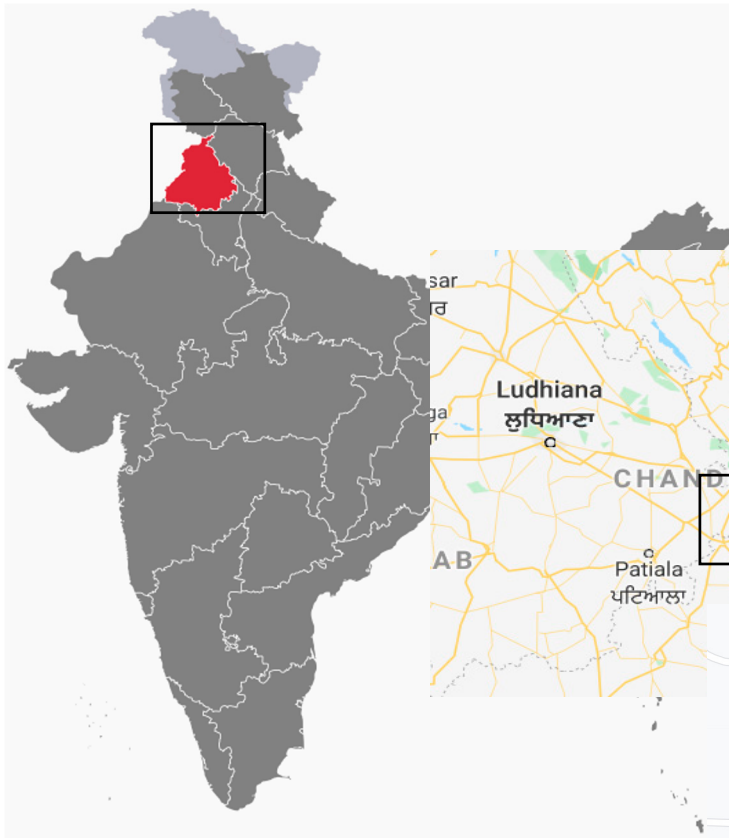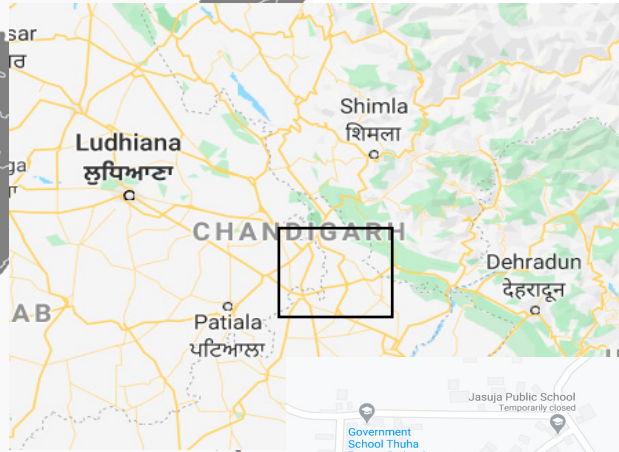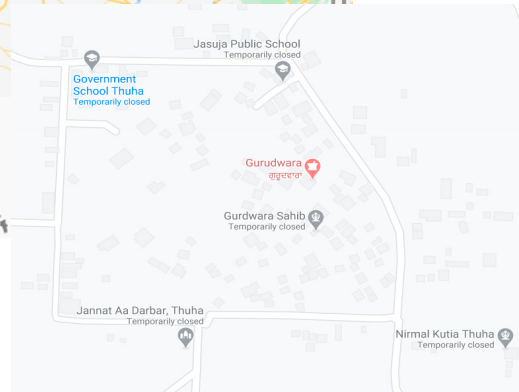

Supplement: Supplementary file 1 [file ijerph-18-04851-s001.zip › supplementary file2.pdf]
